# Total Reference Air Kerma is Associated with Late Bowel Morbidity in Locally Advanced Cervical Cancer Patients Treated with Image-Guided Adaptive Brachytherapy

**DOI:** 10.3390/jcm8010125

**Published:** 2019-01-21

**Authors:** Sophie Bockel, Alexandre Escande, Isabelle Dumas, Elena Manea, Philippe Morice, Sebastien Gouy, Eric Deutsch, Christine Haie-Meder, Cyrus Chargari

**Affiliations:** 1 Brachytherapy Unit, Gustave Roussy, 94800 Villejuif, France; sophie.bockel@gustaveroussy.fr (S.B.); alexandre.escande@gmail.com (A.E.); isabelle.dumas@gustaveroussy.fr (I.D.); dr.elenamanea@yahoo.com (E.M.); eric.deutsch@gustaveroussy.fr (E.D.); christine.haiemeder@gustaveroussy.fr (C.H.-M.); 2 Radiotherapy Department, Gustave Roussy, 94800 Villejuif, France; 3 Radiotherapy Department, Oscar Lambret Comprehensive Cancer Center, 59000 Lille, France; 4 Surgery Department, Gustave Roussy, 94800 Villejuif, France; philippe.morice@gustaveroussy.fr (P.M.); sebastien.gouy@gustaveroussy.fr (S.G.); 5 Institut de Recherche Biomédicale des Armées, 91180 Bretigny-sur-Orge, France; 6 French Military Health Academy, Ecole du Val-de-Grâce, 75015 Paris, France; 7 Brachytherapy unit, Radiation Oncology Department, Gustave Roussy Cancer Campus, 114 rue Edouard Vaillant, 94800 Villejuif, France

**Keywords:** cervical cancer, total reference air kerma, dose-volume parameters, image-guided adaptive brachytherapy, morbidity, normal tissue complication probability, bowel, sigmoid

## Abstract

No dose volume parameter has been identified to predict late bowel toxicities in locally advanced cervical cancer (LACC) patients treated with image-guided adaptive brachytherapy. We examined the incidence of bowel toxicities according to the total reference air kerma (TRAK) in 260 LACC patients. In both univariate and multivariate analysis, late morbidity positively correlated with a TRAK ≥2 cGy (centigray) at 1 meter, emphasizing the importance of this parameter in term of late bowel morbidity. **Objective:** There is no validated dose volume parameter to predict late bowel toxicities in cervical cancer patients treated with image-guided adaptive brachytherapy (IGABT). We examined the incidence of bowel toxicities according to the TRAK, which is proportional to the integral dose to the patients. **Material/Methods:** Clinical data of 260 LACC patients treated with curative intent from 2004 to 2016 were examined. Patients received chemoradiation plus a pulse-dose rate IGABT boost. The relationship between TRAK and morbidity was assessed by Kaplan-Meier method, log-rank tests, and Cox proportional-hazards model on event-free periods. **Results:** Median follow-up was 5.2 years (SE (Standard Error): 0.21). Probability of survival without late bowel toxicity Grade ≥ 2 rate for patients without recurrence (*n* = 227) at 5 years was 66.4% (SE 3.7). In univariate analysis, bowel and/or sigmoid dose/volume parameters were not significant. Late morbidity positively correlated with active smoking, CTVHR volume >25 cm^3^, and a TRAK ≥2 cGy at 1 meter. In multivariate analysis, the following factors were significant: Active smoking (*p* < 0.001; HR: 2.6; 95%CI: 1.4–5.0), and the TRAK (*p* = 0.02; HR: 2.4; 95%CI: 1.2–5.0). **Conclusion:** TRAK was associated with late bowel toxicities probability, suggesting that the integral dose should be considered, even in the era of IGABT.

## 1. Introduction

The standard definitive treatment for locally advanced cervical cancer (LACC) relies on chemoradiation followed by a brachytherapy boost [1,2,3]. The implementation of image-guided adaptive brachytherapy (IGABT) has yielded to an increased capability to perform isodose optimization, allowing dose escalation to the clinical target volumes (CTV) while ensuring a limited dose to organs at risk (OARs), which can be accurately delineated for dose volume histograms (DVH) analysis.

In the last International Commission on Radiation Units (ICRU) guidelines for cervical cancer brachytherapy, consensual OARs dose/volume parameters have been proposed for treatment optimization [4]. Doses delivered to OAR in small volumes through D_0.1 cm^3^_ and D_2 cm^3^_ should be reported [5,6]. From large retrospective and prospective studies, clear relationships have been established between these dose/volume parameters and the probability of occurrence of late rectal or genitourinary morbidity [7,8].

Despite advances in external beam radiotherapy (EBRT) techniques, including intensity-modulated radiotherapy (IMRT) and image-guided radiotherapy (IGRT), bowel toxicity remains a major issue after pelvic irradiation [9]. The reported incidence of all grades late gastro-intestinal toxicities ranges from 8% to 50% in cervical cancer patients and in rare cases a surgical approach might be required to palliate the most severe complications [10,11,12,13,14]. To date, no single dose-volume effect has been shown to predict late bowel or sigmoid toxicity. In institutional studies examining bowel morbidity issue, through a precise delineation of the bowel loops close to the CTV, no significant dose volume effect relationship could be evidenced between the D_2 cm^3^_ and D_0.1 cm^3^_ and the probability of late bowel morbidity [15,16,17]. One hypothesis is that the mobility of bowel makes its dose/volume parameters highly uncertain and the doses reported at time of image acquisition may therefore not reflect the actual dose delivered [18,19,20]. Another hypothesis is that most frequent bowel morbidities (e.g., diarrhea) may be dependent on low doses regions [12,21,22].

The total reference air kerma (TRAK) is the sum of the products of the Reference Air Kerma Rate and the irradiation time for each source. It is directly proportional to the integral dose to the patients. Recent data showed that the TRAK predicted isodose surface volumes in cervix cancer IGABT [23]. This study aims to examine correlation between the TRAK and late bowel toxicities in a large homogenous cohort of patients treated with pulse dose rate (PDR)-IGABT. 

## 2. Materials and methods

### 2.1. Patients

Clinical data of LACC patients treated with curative intent in our institution were retrieved. All patients had a pelvic magnetic resonance imaging (MRI) and a Positrons Emission Tomography/Computed Tomography (PET/CT) as part of their primary staging. In case no para-aortic lymph nodes uptake was obvious on PET/CT, patients without contra-indication underwent a laparoscopic paraaortic lymph node dissection (PALND) to determine the extent of radiation fields [24]. This retrospective study was conducted in accordance with the ethical standards of the 1964 Helsinki declaration and its later amendments and approved by the institutional review board for gynecological cancers.

### 2.2. Treatments

All patients were treated with conformal EBRT or intensity-modulated radiation therapy (IMRT) technique, delivering a total dose of 45 Gy using standard fractionation of 1.8 Gy per daily fraction. Clinical target volumes (CTV) were delineated according to the recommendation and planning target volume (PTV), was generated as previously described [15,25].

Para-aortic irradiation was delivered in case of PET positive para-aortic lymph node metastases or in case of PALND showing presence of para-aortic nodal metastases. Nodal boosts to macroscopic lymph node metastases were delivered sequentially or with simultaneous integrated boost (SIB) technique, in order to achieve a total dose of 60 Gy, taking brachytherapy contribution into account. Concomitant chemotherapy (weekly cisplatin, 40 mg/m^2^) was delivered in patients with sufficient kidney and bone marrow function. In case of renal failure, weekly carboplatin Area Under the Curve (AUC) 2 was used. A PDR MRI-guided BT (Brachytherapy) boost was delivered few days following the last EBRT fraction, in order to keep overall treatment time < 55 days [26]. PDR BT boost was delivered through continuous hourly pulses, with pulse size chosen to keep the D_2 cm^3^_ dose rate less than 0.6 Gy per hour.

A personalized vaginal mould applicator was used in nearly all cases, as previously described [27]. In case parametrial invasion was too important for optimal tumor coverage by endocavitary application only, an interstitial application based on ovoids and needles was performed, either in the same procedure or as a second implant. Brachytherapy treatment planning was based on an MRI in T2 sequence: Axial, sagittal, and coronal slices were acquired [26]. In case of refusal or contraindication to MRI, a CT scan (3 mm slices thickness) was done with intra-venous iodine injection. Images were transferred to treatment planning system. The applicator was reconstructed and the volumes of interest were delineated according to current guidelines: High-risk CTV (CTV_HR_) and intermediate-risk CTV (CTV_IR_), and OARs, including the sigmoid colon, and bowel loops [5]. The sigmoid structure was defined as the outer sigmoid wall, delineated from the recto-sigmoid flexure to at least 2 cm above the parametria and the uterus. The bowel structure was defined as the outer contours of the loops positioned within 3–4 cm to the uterus and applicator, as recommended in EMBRACE study [28]. 

BT aimed at delivering a total dose of 85Gy to 90% (D_90_) of the CTV_HR_, and at least 60 Gy to 90% (D_90_) of the CTV_IR_ (doses in 2-Gy equivalents, EQD2, summing EBRT and BT and applying the linear quadratic model with an α/β ratio of 10 Gy and a half-time repair of 1.5 hours). Dose constraints were 75 Gy to the D_2 cm^3^_ of the sigmoid colon, (EQD2, similar model with α/β = 3 Gy). The TRAK was reported, as well as the treated volume (the volume encompassed by the 15 Gy isodose).

### 2.3. Follow Up

MRI and clinical examination were performed six to eight weeks after the treatment completion. Patients were then evaluated clinically every three months during 2 years, then every 6 months after treatment completion. An MRI was performed every 6 months. Four types of gastro-intestinal events were reported: Diarrhea, flatulence, bowel or sigmoid fistula, and bowel or sigmoid stenosis. These items were chosen according to previous publication examining bowel morbidity from EMBRACE (Image guided intensity modulated External beam radiochemotherapy and MRI based adaptive BRAchytherapy in locally advanced CErvical cancer) study [28]. Late bowel morbidity, defined as a gastro-intestinal toxicity event occurring or lasting over 90 days after treatment completion, was evaluated using the Common Toxicity Criteria for adverse events, version 4.0.

Patients with persistent disease after completion of the treatment or who experienced local relapse were not eligible for morbidity assessment.

### 2.4. Statistical Analysis

Potential predictive factors of bowel toxicity were examined from medical charts, and included clinical variables, as well as detailed treatment related variables (dosimetric parameters, including the TRAK). In case of two fractions, dosimetric parameters of each fraction, including the TRAK, were summed. 

Toxicity-free survival was calculated from the time of treatment completion using Kaplan Meier method. Impact of factors on toxicity-free survival was assessed using Log rank Test or Cox regression model. Both proportional and linear hazards assumptions were validated before analysis. In multivariate analysis, only non-redundant factors with *p*-value < 0.1 were considered. In case of linearity failure, variables were dichotomized depending on results from literature and interquartile. A *p*-value below 0.05 was considered as statistically significant. Statistical analyses were carried out using R studio, version 3.3.3 (RStudio Team (2016). RStudio: Integrated Development for R. RStudio, Inc., Boston, MA, USA).

## 3. Results

### 3.1. Patients

A total of 260 patients treated from 2004 to 2016 were included for analysis. The median follow-up was 5.2 years (SE 0.210). Median age was 48 years (Interquartile IQ (41.2–55.3)). A total of 45 patients (17.4%) had a past history of chronic disease, such as chronic hypertension, diabetes, or systemic disease, and 82 patients (31.8%) were active smokers at time of treatment (Table 1). 

### 3.2. Treatments

Of the 260 patients, 60% (*n* = 156) had laparoscopic staging performed and 93.4% (*n* = 241) received concomitant chemotherapy, median number of 5 cycles. A total of 108 patients (41.5%) received a nodal boost. Thirty-one patients (12%) received para-aortic irradiation to para-aortic region. IMRT was used in 37 patients (14.2%), and 223 patients (85.8%) received 3D conformal EBRT. In 20 cases, interstitial brachytherapy was performed. Median CTV_HR_ was 48 cm^3^ in patients receiving interstitial implantation, *versus* 22 cm^3^ in patients treated with intracavitary brachytherapy only (*p* < 0.001). Treatment characteristics are summarized in Table 1.

**Figure 1:**
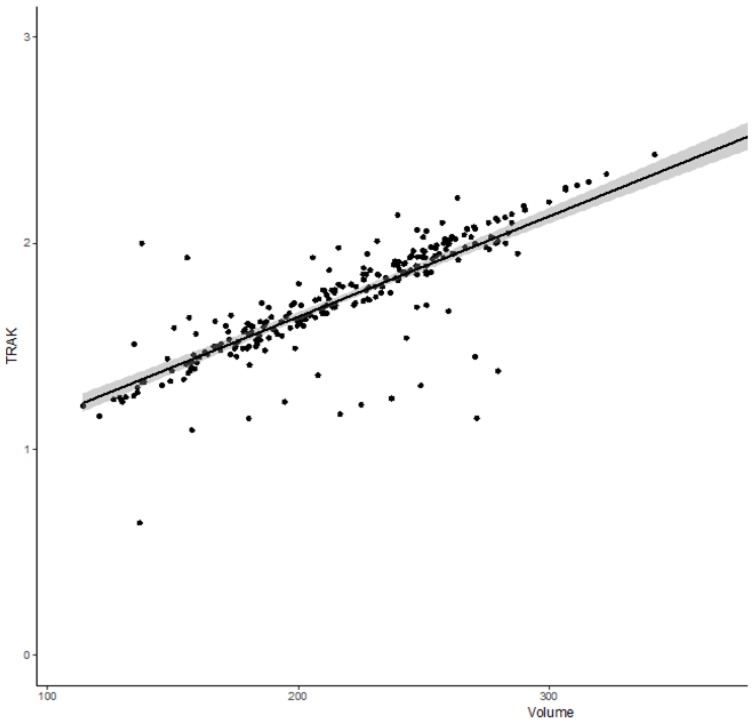
Correlation curve between total reference air kerma (TRAK) and the volume encompassed by 15 Gy isodose volume (*p* < 10^−5^, rho = 0.84).

The median sigmoid D_0.1 cm^3^_ and D_2 cm^3^_ were 63.3 Gy_EQD2_ (IQ: 51.6–72.5) and 56 Gy_EQD2_ (48.2–61.4), respectively. The median bowel D_0.1 cm^3^_ and D_2 cm^3^_ were 76.1 Gy_EQD2_ (IQ: 62.7–95.3) and 61.4 Gy_EQD2_ (IQ: 54.6–71.1), respectively. Median TRAK was 1.73 cGy at 1 m (IQ: 1.54–1.94). The median 15 Gy isodose volume, which represents the treated volume, was 216 cm^3^ (IQ: 182–250). There was a significant but moderate correlation between larger CTV_HR_ volume and interstitial brachytherapy use (*p* < 0.001, rho = 0.23), and between larger CTV_HR_ volume and higher TRAK (*p* < 0.001 and rho = 0.6). No significant correlation between TRAK and interstitial BT use was found. There was a highly significant and very strong correlation between the TRAK and the volume encompassed by the reference isodose surface volume (15 Gy isodose) (*p* < 10^−5^, rho = 0.84) (Figure 1).

### 3.3. Toxicity 

**Figure 2:**
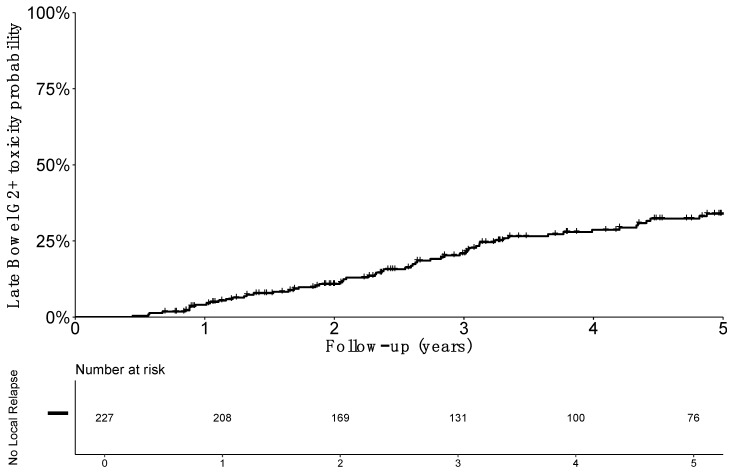
Estimated probability of grade 2 or more (G2+) late bowel toxicity incidence among patients without local relapse.

After exclusion of patients with local failure (including persistent disease), 227 patients were assessable for bowel toxicity (detailed in Table 2). A total of 33 patients (14.5%) reported bowel treatment-related toxicity Grade ≥ 2 during the follow-up, with 30 (12.2%) grade 2 and only three cases (1.3%) of grade 3. No grade ≥ 4 late bowel toxicity was reported. Diarrhea and flatulence grade ≥2 were the most frequent events with 20 patients (8.8%) and 20 patients (8.8%) experiencing each of these events. Fistula and stenosis occurred in two and one patient, respectively. Probability of survival without grade ≥2 late bowel toxicity for patients without recurrence (*n* = 227) at 1, 3, and 5 years were, respectively, 96.8% (SE 1.3), 79.5% (SE 2.9), and 66.4% (SE 3.7), respectively (Figure 2).

### 3.4. Factors Associated with Gastrointestinal Morbidity

Results of univariate analysis for bowel morbidity are reported in Table 3. No significant correlation was shown between sigmoid and bowel dose volume parameters and the probability late toxicity incidence: With p-values of 0.39 (HR: 1.2; 95%CI: 0.6–2.6) and 0.79 (HR: 1.1; 95%CI: 0.5–2.3) for a sigmoid D_2 cm^3^_ > 60 Gy_EQD2_ and D_0.1 cm^3^_ > 70 Gy_EQD2_, respectively, and with p-values of 0.57 (HR: 0.8; 95%CI: 0.4–1.7) and 0.45 (HR: 0.7; 95%CI: 0.4–1.5), respectively for a bowel D_2 cm^3^_ > 65 Gy_EQD2_ and D_0.1 cm^3^_ > 70 Gy_EQD2_.

There was also no correlation between probability of late bowel morbidity and the following clinical factors evaluated: Age > 65 years (HR: 1.0; 95%CI: 0.3–3.3), chronic disease (HR: 1.1; 95%CI: 0.5–2.8), para aortic lymph node dissection (HR: 1.3; 95%CI: 0.6–2.7), para-aortic irradiation (HR: 1.3; 95%CI: 0.6–2.8), IMRT (HR: 0.4; 95%CI: 0.1–1.9), or the use of interstitial brachytherapy (HR: 1.590; 95%CI: 0.7–3.5). Nodal boosts delivery did not reach significance either (*p* = 0.06; HR: 2.0; 95%CI: 1.0–3.9)

Conversely, the following independent factors were found significantly associated with the probability of late bowel morbidity: Active smoking at time of treatment (*p* < 0.005; HR: 2.7: 95%CI: 1.4–5.3), a CTV_HR_ volume >25 cm^3^ (*p* = 0.03; HR: 2.1; 95%CI: 1.1–4.2), and a TRAK ≥ 2 cGy at 1 m (*p* = 0.01; HR: 3.4; 95%CI: 1.6–7.2). The association remained significant in multivariate analysis for the following factors: Active smoking (*p* < 0.001; HR: 2.6; 95%CI: 1.3–5.1), and a TRAK ≥ 2 cGy at 1 m (*p* = 0.03; HR: 2.6; 95%CI: 1.1–6.2). 

**Figure 3:**
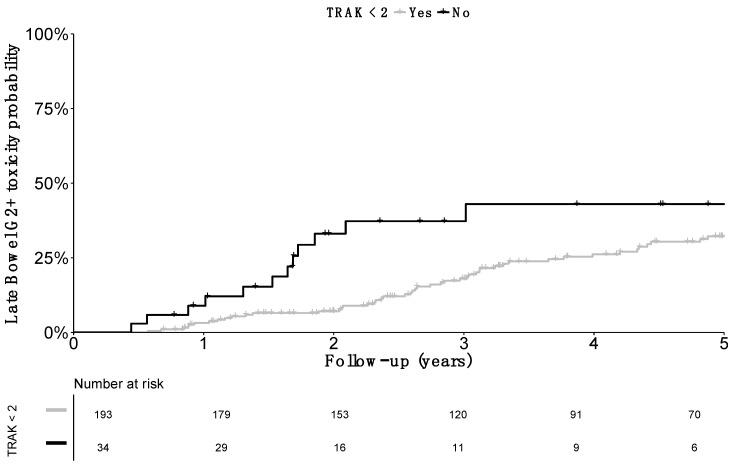
Cumulative incidence of grade 2 or more (G2+) late bowel toxicity cumulative incidence according to the Total Reference Air Kerma. Comparison was significant (*p* value = 0.01).

The impact of TRAK was more specifically assessed: cumulative probability of grade≥2 late bowel morbidity was 6.1% (SE 4.2) at 1 year, 35.3% (SE 9.1) at 3 years, and 35.3% (SE 9.1) at 5 years in patients with a TRAK ≥ 2 cGy at 1 meter. Probability was decreased to 2.6% (SE 1.2) at 1 year, 11.2% (SE 2.5) at 3 years, and 15.5% (SE 3.1) at 5 years in patients with a TRAK < 2 cGy at 1 meter (Figure 3).

## 4. Discussion

Gastro-intestinal toxicity remains a major issue in radiation therapy, even with modern techniques. Late symptoms, even of low grade, may have a major detrimental effect in long term survivors [29,30].

To our knowledge, the present study is the largest single center cohort assessing factors associated with late bowel morbidity in LACC patients receiving chemoradiation plus IGABT. With long-term follow-up, 38 patients (16.8%) experienced grade ≥ 2 late bowel treatment-related toxicity during the observation period. In a prospective study, Chopra et al. found that grade ≥ 2 and grade ≥ 3 bowel toxicity was seen in 30.9% and 12.6% of the patients, respectively [12]. Other retrospective data have reported diarrhea in more than 50% of patients, with 18.4% reporting signs of intermittent subacute bowel obstruction [10]. In the recently published analysis of physician- and patient-reported bowel morbidity from multicenter prospective EMBRACE study, incidence of grade 3–4 events at 3 years was 5%, and grade 1–2 morbidity was reported as 28% [28]. These disparities between studies could be partly explained because the incidence of chronic bowel symptoms is difficult to measure [30]. Differences in the radiotherapy techniques may also explain the relatively good functional outcome reported here, as compared to other data from literature. First, by a primary staging strategy based on PALN dissection, prophylactic para-aortic irradiation was avoided. Second, the whole pelvis dose was always kept at 45 Gy, *versus* 50 Gy in other literature studies [17]. 

While published dose–volume constraints for limiting EBRT-induced gastro-intestinal toxicity exist [8], no dose–volume effect relationship for bowel or sigmoid has been established with the use of IGABT, despite the substantial delivery of radiation dose to the bowel. Similarly, our study failed to show any relationship between bowel or sigmoid D_0.1 cm^3^_ and D_2 cm^3^_ and the occurrence of late bowel toxicity [15]. One potential explanation may rely on the difficulty to establish reliable dose/volume parameters, because of the high mobility of the bowel and sigmoid loops, the dose being delivered to a different region of the bowel or of the sigmoid from one pulse or one fraction to another [16,17,18,19].

In our experience, a TRAK ≥ 2 cGy at 1 m was associated with higher probability of late gastro-intestinal toxicity grade ≥ 2. Scarce data from 2D brachytherapy had suggested that the TRAK could be associated with the probability of complication [31]. However, this association between bowel toxicity and integral dose is a novel finding in the era of 3D optimization. The ICRU/GEC-ESTRO (International Commission on Radiation Units and Measurements/Groupe Européen de Curiethérapie-European Society for Radiotherapy & Oncology)) 89 report recommends the TRAK to be reported as an important dosimetric parameter. TRAK, a simple and unambiguous quantity, is defined as the integral of the reference air kerma rate from all sources at a distance of 1 m from the source over the treatment duration [4]. The concept of isodose surface volumes, which is the volume enclosed by a specific isodose in terms of a physical dose or a biological equivalent dose in EQD2, has been also introduced [4]. In a recent study, Nkiwane et al. showed that there was a specific relation between TRAK and irradiated volume, which is valid across different applicators, dose rates, and fractionation schedules. As a result, the TRAK represents overall treatment intensity in terms of irradiated volume [23]. This very close correlation between the TRAK and the irradiated volume was confirmed in our study. The fact that an increasing TRAK, but not small bowel and sigmoid D_0.1 cm^3^_ and D_2 cm^3^_, was associated with a higher probability of late bowel toxicity grade ≥ 2 suggests that low to intermediate doses delivered in large volume could have a significant impact in the pathogenesis of late bowel toxicity. In fact, the TRAK is a good surrogate of the dose delivered in the patient at 10 cm from the sources, and therefore reflects the integral dose delivered to digestive organs.

Unlike other studies ([32]) in our analysis, performing a para-aortic irradiation was not associated with more frequent grade ≥ 2 bowel morbidity, probably partly because of the low number of patients undergoing para-aortic irradiation (11.9%). The effect of nodal boost delivery was also not significant (*p* = 0.06). The lack of significance of IMRT use is probably related to the low number of patients treated with this technique in our series, while recent randomized studies have shown that IMRT was associated with less gastro-intestinal toxicity, as compared to conventional EBRT [33,34]. A recently published study confirmed that the integration of IMRT may improve acute bowel tolerance to pelvic radiation treatment, as assessed by patient-reported measures. Long-terms results are however awaited to ensure that the beneficial effect is maintained over time [34]. 

Finally, we observed that smoking was associated with higher probability of late bowel toxicity grade ≥ 2 (HR: 2.6; 95%CI: 1.3–5.1). This is consistent with the study published by Eifel et al., in which heavy smoking was the strongest independent predictive factor of overall complications (multivariate HR: 2.30; 95%CI: 1.84–2.87) and particularly of bowel morbidity (HR for smokers of one or more packs per day: 3.25; 95%CI: 2.21–4.78) [35].

Biases inherent to the retrospective analysis should be recognized. First, we were unable to examine the effect of smoking more thoroughly (e.g., number of cigarettes per day, duration of smoking), as well as the effect of other risk co-factors. Moreover, there might be some reporting biases, and the true incidence of low grade bowel toxicities may be underestimated in this cohort. Another limitation is that we examined the effect of TRAK only for PDR treatment planning, and therefore the effect may be somewhat different in high-dose rate (HDR) treatments. In part due to the use of PDR allowing for radiobiological optimization, we had a low use of interstitial needles in this population. However, the TRAK increase may partially reflect a trend to increase overall irradiation volume to achieve an appropriate coverage of parametrial disease, as shown by the significant correlation between CTV_HR_ volume and the TRAK. We also found a significant correlation between CTV_HR_ volume and interstitial brachytherapy use (*p* < 0.001, rho = 0.23), but no significant correlation between TRAK and interstitial use. This suggests that interstitial brachytherapy has an important place, not only to decrease the dose to OARs in the context of dose escalation (as previously published), but also in order to keep the irradiated volume as low as possible [36].

The next step will be to more thoroughly examine how the TRAK could be predictive of toxicities in HDR patients and in independence, in order to potentially derive a bio-TRAK allowing comparisons of both treatment modalities. This is a work in progress in EMBRACE studies [37]. The true contribution of EBRT dose/volume parameters should also be more thoroughly examined, as there may be interactions between the dosimetric improvements afforded by IMRT use and the correlation between TRAK and late bowel morbidity probability. Finally, the relevance of the TRAK as a soft or hard constraint to be considered in treatment planning process should be weighed against the low incidence of severe complications (too low incidence for any statistical analysis in our cohort), and the very poor prognosis of local relapse in these patients.

## 5. Conclusions

We found a significant correlation between the TRAK (and therefore the irradiated volume) and the probability of late bowel morbidity, suggesting the importance of this parameter in term of late bowel toxicity analysis in patients treated with PDR-IGABT. The pertinence of applying a constraint for TRAK in the treatment planning process should be further evaluated in prospective independent cohorts

## Figures and Tables

**Table 1:** Patients, tumours, and treatment characteristics (*n* = 260).

Patients	Performans Status	*n* (%) or Median (IQ)
Age (at diagnosis, in years)		48.0 (41.2–55.3)
Chronic disease *		45 (17.4%)
Active tobacco use		82 (31.8%)
Performance Status		0 (0–1)
	0	133 (51.1%)
	1	109 (41.9%)
	2	18 (7.0%)
Tumors		
FIGO	IB1-IIA	79 (30.4%)
	IIB	112 (43.1%)
	IIIA-IVA	38 (14.6%)
	IVB	31 (11.9%)
Treatments		
Para-aortic lymph node dissection		156 (60.0%)
Overall treatment time (days)		48 (44–52)
Extended field radiotherapy		31 (12%)
Nodal boost delivery		108 (41.5%)
External beam radiotherapy technique		
	IMRT	37 (14.2%)
	3D conformal radiotherapy	223 (85.8%)
Chemotherapy		241 (93.4%)
Number of cycles		5 (4–5)
	<3	(0.8%)
	3	7 (2.7%)
	4	57 (22.1%)
	5	166 (64.3%)
	6	11 (4.3%)
Cisplatin use		210 (81.4%)
Brachytherapy parameters		
CTV_HR_ volume (cm^3^)		22.5 (16.4–32.8)
CTV_IR_ D90 (Gy_EQD2_)		68.2 (65.2–71.2)
CTV_HR_ D90 (Gy_EQD2_)		80.9 (73.9–88.4)
15 Gy isodose volume (cm^3^)		216 (182–250)
Sigmoid D_0.1 cm^3^_ (Gy_EQD2_)		63.3 (51.6–72.5)
Sigmoid D_2 cm^3^_ (Gy_EQD2_)		56.0 (48.2–61.4)
Bowel D_0.1 cm^3^_ (Gy_EQD2_)		76.1 (62.7–95.3)
Bowel D_2 cm^3^_ (Gy_EQD2_)		61.4 (54.6–71.1)
TRAK (cGy at 1 meter)		1.73 (1.54–1.94)
Interstitial brachytherapy use		20 (7.7%)

* Chronic diseases defined as: high blood pressure, dyslipidemia, vascular disease, connective tissue disease, sarcoidosis, diabetes mellitus, obesity. D90 CTV_HR_: minimal dose delivered to 90% of high risk clinical target volume; D90 CTV_IR_: minimal dose delivered to 90% of intermediate risk clinical target volume; FIGO: international federation of gynecology, obstetrics; IMRT: intensity modulated radiotherapy; IQ: interquartile; TRAK: total reference air kerma.

**Table 2:** Patterns of late gastro-intestinal toxicity among patients without relapse (*n* = 227).

Toxicity Patterns/Grade	*n* (%) or Median (IQ)
Grade 2+	33 (14.5%)
Grade 3+	3 (1.3%)
Diarrhea	20 (8.8%)
Grade 2	19 (8.4%)
Grade 3	1 (0.4%)
Flatulence	20 (8.8%)
Grade 2	20 (8.8%)
Stenosis of sigmoid	1 (0.4%)
Grade 2	1 (0.4%)
Fistula of sigmoid	2 (0.8%)
Grade 3	2 (0.8%)

**Table 3:** Univariate factors associated with grade 2+ late bowel toxicity.

		*p* Value	HR	(95%CI)
Age > 65 years		0.99	1.0	(0.3–3.3)
Tobacco use		0.00	2.7	(1.4–5.3)
Chronic disease *		0.81	1.1	(0.5–2.8)
Chemotherapy		0.62	1.7	(0.2–12.1)
CTV_HR_ volume > 25 cm^3^		0.03	2.1	(1.1–4.2)
Nodal boost irradiation		0.06	2.0	(1.0–3.9)
Para-aortic lymph node dissection		0.47	1.3	(0.6–2.8)
IMRT use		0.27	0.4	(0.1–1.9)
Interstitial brachytherapy use		0.24	1.6	(0.7–3.5)
Sigmoid D_0.1 cm^3^_				
	>70 GyEQD2	0.79	1.1	(0.6–2.6)
	>65 GyEQD2	0.94	1.0	(0.5–2.0)
Sigmoid D_2 cm^3^_				
	>60 GyEQD2	0.39	1.2	(0.6–2.6)
	>55 GyEQD2	0.50	0.8	(0.4–1.5)
Bowel D_0.1 cm^3^_				
	>75 GyEQD2	0.96	1.0	(0.5–2.0)
	>70 GyEQD2	0.45	0.8	(0.4–1.5)
Bowel D_2 cm^3^_				
	>70 GyEQD2	0.76	0.9	(0.4–1.9)
	>65 GyEQD2	0.57	0.8	(0.4–1.7)
TRAK	≥1.8	0.01	2.6	(1.2–5.3)
TRAK ≥ 2.0		0.01	3.4	(1.6–7.2)

HR: hazard ratio; CI: confidence interval; TRAK: Total Reference Air Kerma; EQD2: dose equivalent per 2-Gy fractions; IMRT: intensity modulated radiotherapy. * Chronic diseases defined as: high blood pressure, dyslipidemia, vascular disease, connective tissue disease, sarcoidosis, diabetes mellitus, obesity.
